# High-resolution label-free 3D mapping of extracellular pH of single living cells

**DOI:** 10.1038/s41467-019-13535-1

**Published:** 2019-12-06

**Authors:** Yanjun Zhang, Yasufumi Takahashi, Sung Pil Hong, Fengjie Liu, Joanna Bednarska, Philip S. Goff, Pavel Novak, Andrew Shevchuk, Sahana Gopal, Iros Barozzi, Luca Magnani, Hideki Sakai, Yoshimoto Suguru, Takuto Fujii, Alexander Erofeev, Peter Gorelkin, Alexander Majouga, Dominik J. Weiss, Christopher Edwards, Aleksandar P. Ivanov, David Klenerman, Elena V. Sviderskaya, Joshua B. Edel, Yuri Korchev

**Affiliations:** 1 Department of Medicine, Imperial College London, London, W12 0NN UK; 2 Tianjin Neurological Institute, Tianjin Medical University General Hospital, Tianjin, 300052 China; 3 Nano Life Science Institute (WPI-NanoLSI), Kanazawa University, Kakuma-machi, Kanazawa, 920-1192 Japan; 4 Precursory Research for Embryonic Science and Technology (PRESTO), Japan Science and Technology Agency (JST), Saitama, 332-0012 Japan; 5 Department of Surgery and Cancer, Imperial College London, London, W12 0NN UK; 6 Department of Earth Science & Engineering, Imperial College London, London, SW7 2AZ UK; 7 Cell Biology Research Centre, Molecular and Clinical Sciences Research Institute, St George’s, University of London, London, SW17 0RE UK; 8 National University of Science and Technology “MISIS”, Leninskiy prospect 4, 119991 Moscow, Russian Federation; 9 Department of Pharmaceutical Physiology, Graduate School of Medicine and Pharmaceutical Sciences, University of Toyama, Toyama, 930–0194 Japan; 10 Department of Chemistry, Lomonosov Moscow State University, Leninskiye gory 1-3, GSP-1, 119991 Moscow, Russian Federation; 11 Department of Chemistry, Imperial College London, Molecular Science Research Hub, London, W12 0BZ UK; 12 Department of Chemistry, University of Cambridge, London, CB2 1EW UK

**Keywords:** Nanoscale biophysics, Single-molecule biophysics, Chemistry, Nanoscience and technology

## Abstract

Dynamic mapping of extracellular pH (pHe) at the single-cell level is critical for understanding the role of H^+^ in cellular and subcellular processes, with particular importance in cancer. While several pHe sensing techniques have been developed, accessing this information at the single-cell level requires improvement in sensitivity, spatial and temporal resolution. We report on a zwitterionic label-free pH nanoprobe that addresses these long-standing challenges. The probe has a sensitivity > 0.01 units, 2 ms response time, and 50 nm spatial resolution. The platform was integrated into a double-barrel nanoprobe combining pH sensing with feedback-controlled distance dependance via Scanning Ion Conductance Microscopy. This allows for the simultaneous 3D topographical imaging and pHe monitoring of living cancer cells. These classes of nanoprobes were used for real-time high spatiotemporal resolution pHe mapping at the subcellular level and revealed tumour heterogeneity of the peri-cellular environments of melanoma and breast cancer cells.

## Abstract (web summary)

Current methods to measure extracellular pH are often limited in resolution and response times. Here the authors present a label-free nanoprobe, consisting of a zwitterionic nanomembrane at the tip of a nanopipette, which enables high spatiotemporal resolution pH measurements and topography-pH 3D mapping in live cancer cells.

## Introduction

Cell survival requires the maintenance of a relatively constant neutral extracellular microenvironment. Extracellular acidification occurs due to the stimulation of anaerobic glycolysis under tumour and inflammatory circumstances^1^. For example, acidic extracellular microenvironments can promote tumour metastasis and modulate inflammatory responses. Therefore, the precise measurement of local extracellular pH (pHe) is critical for understanding the role of H^+^ in cell activity and in turn, its implications in cancer diagnosis and treatment^2,3^. Measuring the local pHe is also crucial in assessing the extent of tumour invasion, and immune reaction^1,4^. High-resolution pHe mapping can help to better understand the link between pH distribution, cell morphology and cell function. However, the pHe spatial distribution in the cell microenvironment is exceptionally challenging to map, due to the high mobility and rapid diffusion of extracellular protons^5,6^. There is, therefore, a need to develop an agent- or label-free high-resolution method for effective and sensitive monitoring of pHe changes at the single-cell level^7^.

At present, the most commonly used pH probes are based on conventional microelectrodes and are limited by large footprint and slow response times^8^. Alternatively, fluorescence-based pH probes can be used in extracellular space, but with considerable limitations due to high background levels and rapid photobleaching^9^. Uses of Nuclear Magnetic Resonance Imaging and Positron Emission Computed Tomography have also been reported^10^; however, these methods have a low spatial resolution, and their reliance on the distribution of the probes within tissue makes the quantification of pHe challenging^11^.

Recently, to address part of this challenge, we developed polypyrrole-based Field Effect Transistors (FET) at the tip of a nanopipette, which could be positioned in well-defined locations and detect pH changes in aqueous environments^12,13^. However, these probes had slow response times, severely limiting the dynamic mapping of single cells in real-time^12^. The slow response is due to ionic Coulomb blockade, which reduces the rate of hydronium ion diffusion^14,15^. One promising route in overcoming such limitations is in the use of functional zwitterionic nanomembranes with high conductivity and electroactivity^14–16^. An example that we propose to take advantage of is in the drying-mediated self-assembly of a poly-l-lysine/ Glucose oxidase (PLL/GOx) hydrogel, which can be crosslinked by using glutaraldehyde vapour^17^. The positively charged quaternary amines of PLL and negatively charged carboxylic acid residues of GOx facilitates the self-assembly of zwitterion-like membranes, ensuring heightened H^+^ sensitivity. Furthermore, such nanomembranes allow the ion current to flow through the membrane matrix, which can be used for feedback control when coupled to live-cell imaging methods such as Scanning Ion Conductance Microscopy (SICM). Importantly, this allows for high-resolution 3D topographical imaging of living cells^18^.

**Fig. 1:**
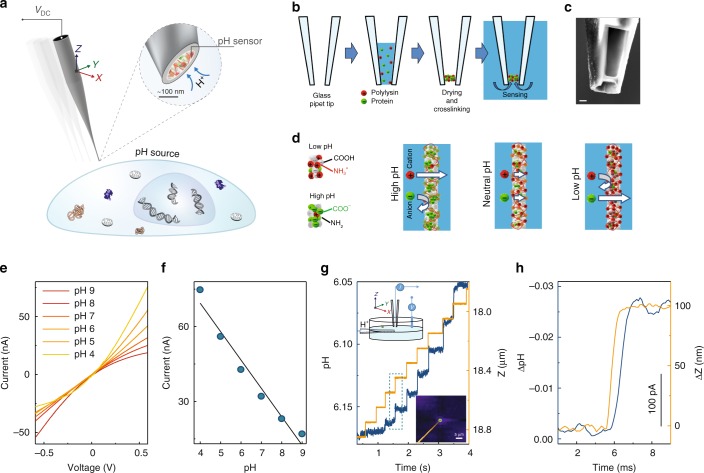
Nanoprobe pH sensor fabrication and characterisation. **a** Conceptual image demonstrating the dynamic mapping of extracellular pH in three dimensions with high spatial resolution. **b** The nanoprobe pH sensors were made by immobilising a mixture of glucose oxidase and poly-l-lysine and made via drying-mediated self-assembly at the tip of a pulled glass nanopipette by crosslinking with glutaraldehyde. **c** A scanning electron microscopy image (scale bar 500 nm) showing the tip of the pH probe consisting of a thin membrane. The pipette tip has been focused ion beam milled using a slice and view technique. **d** Proposed working principle of the sensor, by which the nanomembrane shows preferential permeability for anions at low-pH and cations at high-pH. **e** Current-voltage characterisation of the sensor at varying pH, and **f** current *vs* pH at 0.6 V showing good linear response in the pH range of 4–9 (R^2^ = 0.96, *p* < 0.001, Pearson’s correlation). **g** A nanopipette was used as a highly localised H^+^ source for testing the pH mapping capability of the nanoprobe sensor (left top inset). 2D top view showing the pH distribution profile (right bottom inset) as obtained using SICM mapping. Real-time pH measurements that allow assessing the probe response time and sensitivity. The probe is moved to the H^+^ source in the z-direction using a fast piezo-stage. **h** A magnified plot of the dotted-box shown in **g**, demonstrating the sensitivity and resolution of the nanoprobe pH sensor.

Here we report on the development of a label-free pH-sensitive nanoprobe consisting of a self-assembled zwitterion-like nanomembrane at the tip of a nanopipette. This platform allows for SICM feedback-controlled precise positioning of the nanoprobe to the cell surface to monitor the local pHe with high spatiotemporal resolution and high sensitivity. Furthermore, we show that double-barrel SICM-pH nanoprobes can be fabricated and used to combine the advantages of high-resolution SICM feedback-controlled scanning with high-sensitivity pH-sensing, thus enabling the acquisition of simultaneous topography-pHe 3D mapping of single living cells in real-time, Fig. 1a.

## Results

### Fabrication of a pH-sensitive single-barrel nanoprobe

A schematic of the drying-mediated self-assembly process at the tip of a glass pipette is shown in Fig. 1b and Supplementary Fig. 1. The volatile nature of glutaraldehyde enables the introduction of free aldehyde by vapour diffusion into the membrane matrix and also facilitates the covalent bonding of the crosslinked functional groups^19^. This also leads to the formation of a stable zwitterionic-like nanomembrane, which exhibits ion-current rectification, Fig. 1e and Supplementary Fig. 2c. Such unique zwitterionic properties can be exploited for ultrasensitive pH sensing by controlling the total surface charge of the nanomembrane to prevent the formation of an ionic Coulomb blockade and allow the interfacial H^+^ to be freely and rapidly transferred from the solution phase to the active sensing area^14^. We found that optimal conditions could be achieved by using a ratio of ~0.4 mg/ml GOx to 0.01% PLL, which gives an optimised ion-current rectification as can be seen in the I–V response of the nanoprobe, Supplementary Fig. 2c (blue line). Under these conditions, the nanomembrane shows preferential permeability for anions at low-pH and cations at high-pH, Fig. 1d. An excellent linear response (R^2^ = 0.99, *p* < 0.001) between pH 5 and pH 8 could be obtained, Fig. 1e, f and Supplementary Fig. 2d–f. For physiological applications including sensing of living cells, such improved linear sensitivity is likely to be of greater utility than a wider dynamic range. The properties of these nanomembrane sensors (e.g. surface charge, dynamic range, and pH sensitivity) can be further finely tuned by controlling the GOx to PLL ratio, Supplementary Fig. 2c. GOx was chosen as it is easy to obtain, low cost, has good thermal and pH stability, a long shelf life, and high operational stability. However, other charged proteins/enzymes can also be used such as hexokinase and bovine serum albumin, Supplementary Fig. 3.

### Characterisation of pH-sensitivity

The structural properties of the membrane are crucial in optimising the pH-sensing capability. Slice and view Focused Ion Beam Scanning Electron Microscopy (FIB-SEM) was used to image the tip of the pipette. Direct slice and view imaging was possible since the PLL/GOx nanomembranes were tightly crosslinked. A series of milling cross-sectional FIB-SEM images showed that the PLL/GOx nanomembrane formed at the tip of the pipette is ~200 nm thick, Fig. 1c and Supplementary Fig. 1.

With these probes, ion selectivity can be achieved, in a  similar manner to other types of nanopore sensors, in which the sensing channel opening has comparable dimension to the Debye screening length (nanometre range in physiological solution)^13,20^. The ion selectivity was evaluated by measuring the reversal potential, Supplementary Fig. 2a, b, which was calculated to be between 15–20 mV per pH unit and had an excellent linear response to a change in pH. This high ion selectivity made it possible to operate the nanoprobe in potentiometric mode, Supplementary Fig. 2b, as in a conventional pH sensor.

Under the influence of an electric field, Fig. 1e, f, a six-point calibration was performed between pH 4.0–9.0. Changes in ion-current rectification can be observed in the I–V curves at varying pH and followed a linear response. The linear ionic response can not only be used as a pH indicator, but also as a feedback signal for use in probe-sample distance control for high-resolution 3D imaging when coupled with SICM^18^. Since the spatial resolution of SICM is closely linked to the probe dimensions^18^, the miniaturisation of the nanoprobe is essential to maximising the spatial resolution, Supplementary Fig. 2d–f. For example, at a holding potential of −0.6 V, the ion current flowing through the nanomembrane into the probe ranges from ~−70 nA for a micropipette with ~2.5 µm inner diameter (imaged with SEM in Supplementary Fig. 1) to about −0.8 nA for a nanopipette with a tip inner diameter of ~100 nm as previously investigated by our groups^18,21^.

### Evaluation of SICM feedback-controlled pH sensing

Combined with SICM, the nanoprobe is capable of non-contact surface scanning and accurate positioning at desired locations over a cell or any other H^+^ releasing source. In order to thoroughly investigate the capability of the sensor, we generated an artificial H^+^ gradient using delivery via a voltage-controlled nanopipette, as previously published by our groups^6,21^. This H^+^ gradient generation has the advantage that the voltage could be rapidly switched on or off, allowing us to induce highly localised positive H^+^ release or to perform negative controls in nanoprobe pH mapping experiments, Supplementary Fig. 4a–c. The generated H^+^ gradient can be manipulated by altering the distances from the H^+^ delivery pipette, Fig. 1g. To illustrate the spatial resolution, we carried out 2D X–Y plane pH mapping of an H^+^ supplying nanopipette (~100 nm inner diameter), Fig. 1g and Supplementary Fig. 4a, c. For comparison, a larger pH gradient generated with a voltage-controlled H^+^ supply micropipette were also mapped, Supplementary Fig. 4b.

To evaluate the response time and sensitivity, we used a fast piezo-stage capable of rapidly changing the position of the sensor, Fig. 1g. Response times of ~2 ms could be achieved with a sensitivity better than 0.01 pH units and a spatial resolution higher than 50 nm, Fig. 1f.

The magnitude of the pH gradient depends on cell activity, extracellular buffering and H^+^ diffusion^22,23^. In a non-buffered solution, the high diffusion coefficient enables the protonic charge to travel over 100 µm, whereas, in a physiologically buffered solution the distance travelled can be shortened to about 10 nm^23^. Since physiological media in living organisms are typically buffered solutions, we first investigated the buffering effects on nanoprobe pH sensing using an artificial H^+^ gradient model in solution with different buffering capacity, Supplementary Fig. 5. Proton diffusion is heavily limited and localised with the increasing buffer concentration.

### High-resolution pHe mapping of living cells

**Fig. 2:**
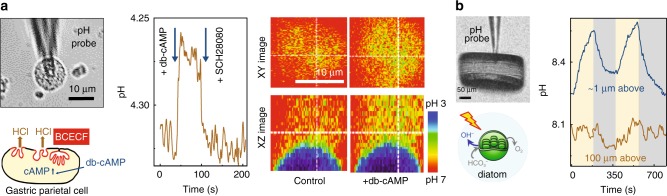
SICM feedback-controlled real-time pHe detection of living cells. **a** Bright filed microscopy image showing  a pH-sensitive nanoprobe over a buffered single gastric parietal cell (left top). The cartoon at the bottom illustrates the construction of extracellular pH measurements (left bottom). Feedback-control allows for positioning of the pH nanoprobe to accurately detect 100 µM db-cAMP (cAMP analogue) triggered via rapid HCl secretion from a single gastric parietal cell. The secretion can be inhibited with selective H^+^, K^+-^ATPase inhibitor SCH28080 (middle). The db-cAMP-induced HCl secretion from the gastric parietal cells was confirmed with pH-sensitive BCECF fluorescent dye by confocal microscopy (right). Changes in fluorescent intensity of pH-sensitive dye can be mapped out either by x–y or by x–z scanning. The dotted crossline marks the Z position in the XZ image and the Y position in the XY image, respectively. **b** pHe detection from a single low-buffered *Coscinodiscus wailesii* cell. Changing light conditions from illumination (yellow stripes represent light illumination) to darkness (grey stripes represent darkness), and vice versa revealed rapid pH changes ~1 µm above the cell surface. Change in pH is almost undetectable when the probe is 100 µm away from the cell surface.

Parietal cells are responsible for the secretion of hydrochloric acid (HCl) in the stomach^24^. With the help of SICM distance control, our single-barrel nanoprobe pH sensor can be navigated and accurately positioned ~1 µm above the cell surface. It was possible to monitor dbcAMP-induced rapid HCl secretion from a single parietal cell, which can be inhibited with the proton pump inhibitor SCH 28080, Fig. 2a. Similar db-cAMP triggered H^+^ release from a single parietal cell was verified by the acidification of extracellular pH-sensitive BCECF dye using confocal microscopy, Fig. 2a.

Unlike the acidic microenvironment of parietal cells, a significant rise in cell surface pH in algae exposed to light is expected due to photosynthetic uptake of dissolved inorganic carbon^25^. Fluctuations of around 0.3 pH units were observed at 1 µm above the surface of marine diatom *Coscinodiscus wailesii* within 200 s of light exposure, Fig. 2b. No such change in pH could be detected 100 µm away from the cell surface, which was attributed to previous observations that light-induced pH change only occurs within the algal external boundary layer^25^.

In SICM, the probe to sample distance is controlled via the decrease of ionic current flowing through the tip of a standard glass nanopipette, as it approaches the sample surface. As another example, pHe mapping of normal melanocytes is shown where no noticeable pH gradients around the cells were observed, Supplementary Fig. 6a–c. SICM uses ionic current as a feedback-control signal for scanning, which is not only sensitive to approximately one probe radius separation between nanoprobe–cell surface, but also to the extracellular pH changes and can induce ball-like topographical artefact at the tip of the H^+^ supply pipette (dotted-circle highlighted in Supplementary Fig. 6d–g). Although such interference of pH sensing can be partially minimised with constant-height (Supplementary Fig. 6h, i) or feedback-controlled iceberg SICM scanning mode, Supplementary Fig. 7, as will be discussed, this limitation can be overcome with the use of double-barrel probes.

### High-resolution 3D pHe mapping of live cancer cells

**Fig. 3:**
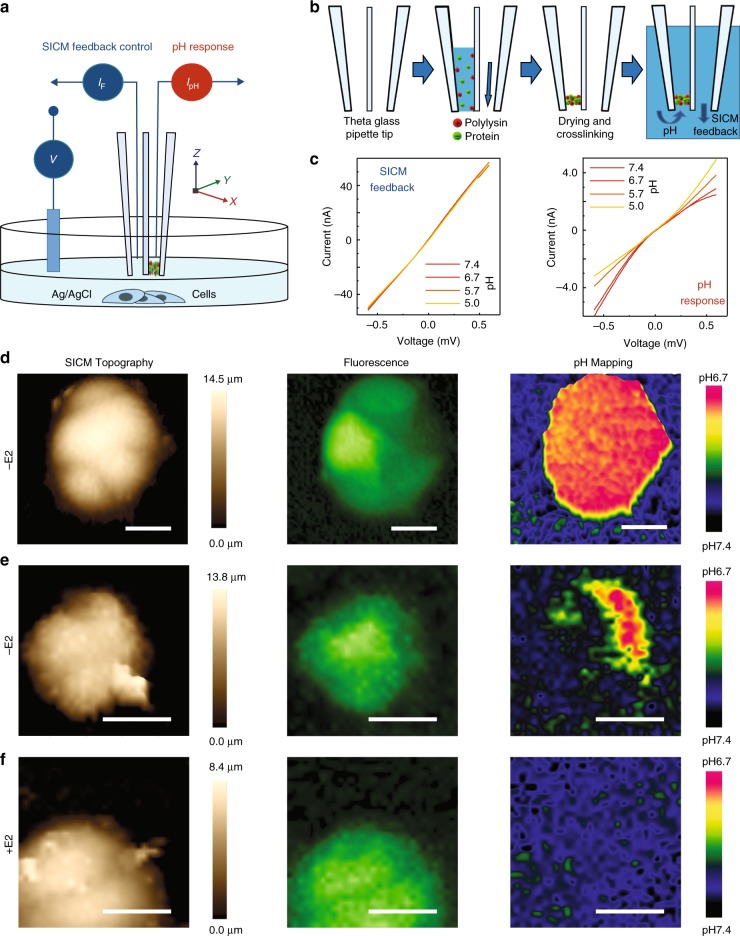
Independent SICM feedback-controlled scanning and simultaneous 3D pHe mapping of living cells. **a** A schematic showing the operation of double-barrel nanoprobe for simultaneous SICM imaging and pH measurement. **b** A pH-sensitive nanomembrane is formed inside one barrel (pH-barrel) of a double-barrel θ quartz glass nanopipette, while the second barrel (SICM imaging -barrel) is kept open via applied back pressure during fabrication. **c** The ion-currents flowing into two separated barrels of the generated double-barrel nanoprobe show different I–V responses to pH. **d** SICM imaging and 3D pHe mapping of a group of low-buffered CD44^GFP-high^ breast cancer MCF7 cells in estradiol-deprived medium (−E2). The SICM topographical images (left), fluorescence image (GFP, middle), and 3D pHe distributions (right) can be simultaneously obtained from a single scan. **e** Same as **d** but using a different group of estradiol-deprived (−E2) CD44^GFP-high^ cells. **f** Same as **d** but using a group of CD44^GFP-high^ cells under estradiol-supplemented culture (+E2). Scale bars represent 20 µm. Intensity of fluorescence images have been normalised.

To decouple the SICM scanning ability from the pH sensing, we fabricated a double-barrel nanoprobe. As demonstrated in the operational (Fig. 3a) and fabrication (Fig. 3b) schematics, the double-barrel SICM-pH nanoprobe consists of an unmodified open barrel (SICM-barrel) for SICM control and another barrel with a pH-sensitive PLL/GOx omembrane (pH-barrel), which enables both pH measurement and SICM topographical imaging simultaneously and independently. The ion-current flowing into the two independent barrels of the double-barrel nanoprobe showed very different I–V responses at varying pH, Fig. 3c. Much like the single-barrel case, the dynamic range, linearity, and sensitivity were similar. In order to measure local pHe accurately, a self-referencing 3D mapping protocol that is used in multifunctional SECM-SICM was employed^26^. Note that such self-referencing measurements allow the response of local pH near to the cell surface (about 100 nm) to compensate for the possible pH drift in bulk (~10 µm over) at every pixel of SICM 3D pH mapping.

We further applied the double-barrel SICM-pH nanoprobe to measure the dynamic change of pH gradients in breast cancer MCF7 cells at the single-cell level, Fig. 3d–f. Previously, it has been demonstrated that CD44^high^ breast cancer cells bear similarity to cancer stem cells and are involved in aggressive phenotypes^27^. Estrogen deprivation therapy is used in the treatment of breast cancer; our previous study demonstrated that only a small fraction of CD44^high^ cells could adapt to such estradiol-deprivation^28^. Using MCF7 cells engineered to drive GFP expression under the promoter of the CD44 gene, estradiol-deprived (−E2) CD44^GFP-high^ cells showed a strong pHe gradient towards acidification, Fig. 3d, as opposed to estradiol-supplemented (+E2) CD44^GFP-high^ cells, which do not generate any noticeable pHe gradient, Fig. 3f. However, pHe mapping across these –E2 CD44^GFP-high^ cells further corroborates the phenotypic heterogeneity in this subpopulation of MCF7 cells, Fig. 3e. This is in line with the heterogeneity in single-cell transcriptomics data, Supplementary Fig. 8, and our previous observations at the transcriptional level^28^.

It has been proposed that thymosin beta-4 × -linked (TMSB4X) may positively regulate the activity of ATP-synthase to transport proton from the intracellular to the extracellular space of cancer cells^29^. Moreover, an ATP-driven, vacuolar proton pump (V-ATPase) has been suggested to be involved in the regulation of extracellular acidification by cancer cells^30^. Using single-cell RNA-sequencing^31^, we found a significantly higher expression of TMSB4X and ATP6V1F in estradiol-deprived (−E2) CD44^GFP-high^ compared to estradiol-supplemented (+E2) CD44^GFP-high^ cells, Supplementary Fig. 8, which may explain the observed difference in pHe, Fig. 3d–f. Adding 10 mM HEPES buffer eliminated most of the observed pHe gradient of these estradiol-deprived (−E2) CD44^GFP-high^ MCF7 cells, Supplementary Fig. 9. Three experiments with continuous repetitive 3D pHe mapping revealed profound effects of buffer on the profile of pH gradients and demonstrated noticeable dynamically changes in pHe, while the changes in topographical and fluorescence images were virtually undetectable, Supplementary Fig. 9. Such buffer treatments may help us to study the dynamic changes in local H^+^ distribution near to cell surface in the future.

**Fig. 4:**
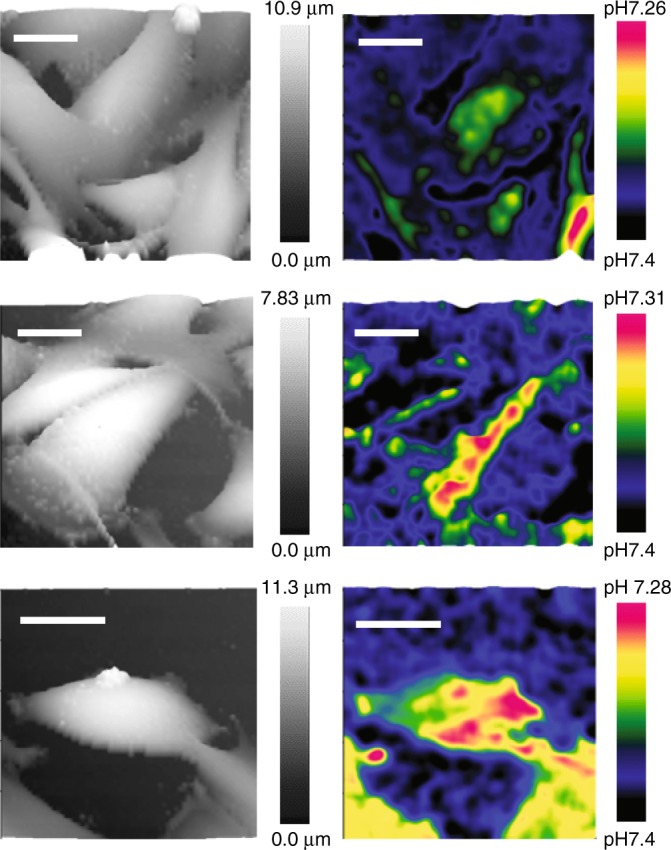
High-resolution 3D pHe mapping of living melanoma cells. The 3D SICM topographical images (left column) and 3D pHe distributions (right column) of three different groups of low-buffered living melanoma A375M obtained simultaneously. Scale bars represent 20 µm.

Since melanoma represents one of the most aggressive and heterogeneous forms of cancer, exhibiting the most diverse cell subpopulations and showing extracellular acidosis^32^, we further applied our nanoprobe for pHe mapping to living melanoma A375M cells, Fig. 4. The high-resolution SICM topographical images (left panel of Fig. 4) and pHe 3D mappings (right panel of Fig. 4) of the same melanoma cells could be obtained simultaneously in a single SICM scan. As expected, high-resolution 3D pHe mapping among these melanoma cells demonstrated a highly variegated distribution pattern, which is in agreement with the most heterogeneous nature of melanoma cells. In contrast, the pHe mapping of non-cancerous melanocytes did not generate a noticeable pHe gradient, Supplementary Fig. 6a–c.

## Discussion

It is becoming clear that an acid pHe plays an essential role in cancer cell progression, invasiveness and resistance to therapy^1,33^. Monitoring the pHe variation of cancer cells is likely to provide critical information for understanding tumour heterogeneity during pathological processes such as epithelial-mesenchyme transition^33^. However, a high diffusion coefficient and heterogeneous distribution of extracellular protons make real-time monitoring of pHe more difficult^34,35^. The development of label-free high-sensitivity high-resolution methods to detect pHe at the single-cell level is urgently needed^7^.

In this study, pH-sensitive nanoprobes have been developed by crosslinking GOx and PLL to produce a drying-mediated self-assembly nanomembrane at the tip of glass nanopipettes, resulting in high spatial resolution of 50 nm, about 2 ms rapid response time, 0.01 pH sensitivity, and with minimal disturbance of the samples. A typical probe can remain functional for ~180 days (dry shelf-life at room temperature) and can be used under varied ionic strength conditions from freshwater to seawater as shown in Supplementary Fig. 10 and Supplementary Fig. 11.

High H^+^ sensitivity of probe is likely due to the pH-dependent modification of the surface charge of the zwitterion-like PLL/GOx nanomembrane^16,36^ or intramolecular interactions among amine and carboxyl groups^37,38^ within PLL/GOx nanomembrane, Fig. 1d. The higher surface-to-volume ratio of the porous nanomembrane may also help the accumulation and adsorption of H^+^ and consequently, the pH sensitivity. Although these hypotheses may explain the sensitivity and precision of our pH-sensitive nanoprobe, further modelling and investigations are still required.

SICM feedback-controlled pH-sensitive single-barrel probes have been used to detect spatiotemporal pHe around a single acid-releasing parietal cell and photosynthesis-induced alkaline of algae. Although the single-barrel probe  demonstrated the possibility to map pHe over a single immune cell or a group of melanoma cells with constant-height or iceberg SICM operational mode, the interference of pH in feedback ion currents made accurate 3D pHe mapping of living cells challenging to achieve. We therefore fabricated a double-barrel SICM-pH nanoprobe to combine pH sensing with robust independent SICM feedback control to perform reliable 3D pHe mapping around living cells. In order to improve the accuracy of local pHe detection around a single cell, a self-referencing approach was introduced to compensate for possible pH drift in bulk solution of the living system^26^. Previous studies have demonstrated that tumour cells can generate 0.2–0.6 units lower pHe gradients than the normal tissues at single-cell level^39^. We detected about 0.7 units lower pHe gradients of a group of estradiol-deprived (−E2) CD44^GFP-high^ breast cancer MCF7 cells (Fig. 3d, e), and about 0.2 units lower pHe gradients of a group of A375M melanoma cells (Fig. 4). Intratumoral heterogeneity has long been recognised as a general characteristic of most cancers, particularly melanoma, which is among the most aggressive and therapy-resistant of human cancers^40^. The spatial resolution of 3D pHe mapping of A375M cells shows a variegated pH gradient pattern (Fig. 4), which may indicate the exceptional level of intratumor heterogeneity and the presence of cell subpopulations with different phenotypes and biological behaviour^32,40^. Such heterogeneous distribution of pHe observed from melanoma cells may also relate to the inhomogeneously distributed proton transporters to the cell border or leading-edge pseudopodia of these invasive cancer cells^41^.

Although single-cell genomics makes it possible to analyse the intratumoral heterogeneity, understanding the cellular heterogeneity of cancer cells is still a big challenge^42^. Using CD44 as a biomarker to select breast cancer stem cells for single-cell sequencing, we have demonstrated that CD44^GFP-high^ breast cancer MCF7 cells consist of multiple subpopulations with marked heterogeneity^28^. In this study, pHe 3D mapping also showed such heterogeneity among CD44^GFP-high^ cells under culture when they were estradiol-deprived (−E2) or estradiol-supplemented (+E2), and even heterogeneity among these estradiol-deprived (−E2) CD44^GFP-high^ aggressive and plastic cell subpopulations (Fig. 3d, e). The single-cell RNA-sequencing showed upregulated gene expressions of extracellular acidification related proteins TMSB4X and V-ATPase^29,30^ (Supplementary Fig. 8), which may partially explain our observed pHe heterogeneity of MCF7 cells. In future work, we plan to assess the heterogeneity of these cancer cells with our double-barrel nanoprobe functional pHe mapping and then select cell subpopulations to perform nanobiopsy sampling from these individual cells^43,44^. This will enable a method for function-driven single-cell sequencing and thus further understanding of cancer heterogeneity, cellular plasticity and therapeutic resistance at the single-cell level.

Extracellular acidic pH enhances the invasive behaviour and the migration of cancer cells and may define an additional barrier for therapy^39^. Normalising the pHe gradient through inhibition of various proton transporters or by systemic buffering with bicarbonate is of therapeutic benefit in cancer treatment^4^. The reported double-barrel SICM-pH nanoprobe self-referencing sensing platform enables real-time feedback-controlled dynamically 3D mapping of pHe heterogeneities of cancer cells label-free and at subcellular resolution. This method could help with a cancer diagnosis, prognosis, and in evaluating acidic pHe targeted therapies.

## Methods

### Chemicals

Poly-l-lysine (PLL, P4707), glucose oxidase (GOx, G2133), HCl and glutaraldehyde were purchased from Sigma–Aldrich. Phosphate buffered saline (PBS) solution was prepared from 7.2 mM Na_2_HPO4, 2.8 mM KH_2_PO_4_, 137 mM NaCl and 2,7 mM KCl (pH 7.4). Hanks’ Balanced Salt Solution (HBSS) containing 5 mM bicarbonate and 0.8 mM phosphate buffer (pH 7.4) was purchased from Gibco.

### Fabrication of the glass pipettes

Single barrel: pH-sensitive nanomembrane probes were fabricated by pulling a borosilicate glass capillary (O.D. 1 mm, I.D. 0.58 mm) to micropipettes with a tip diameter of ~1 µm or nanopipettes with a diameter of ~100 nm using a laser-based puller (Model P-2000, Sutter Instruments Co., USA). Nanopipettes were pulled with a two-step protocol. Briefly, for the initial step, the parameters were: heat 350, filament 3, velocity 30 and delay 200. For the second step, they were: heat 350, filament 2, velocity 27, delay 160 and pull 250. It should be noted that these values are instrument-specific, and parameters would have to be optimised for each instrument in order to obtain similar nanopore.

Double barrel: Double-barrel pH nanoprobes were constructed from a double-barrel quartz theta capillary (O.D., 1.2 mm, I.D., 0.9 mm, Sutter Instruments), which was pulled with a laser-based P-2000 pipette puller (Sutter Instruments) using a single line program (heat 700, filament 3, velocity 45, delay 130, and pull 93) to produce sharp double-barrel nanopipettes. The size of each barrel of this pulled double-barrel nanopipette was about 100 nm.

### Assembly of the pH-sensitive nanomembrane

Glucose oxidase (GOx) has a pI of 4.2 and is therefore negatively charged at physiological conditions. GOx was firstly inactivated by denaturation at 70 °C for 10 min before nanomembrane fabrication. This was then dissolved in 0.01% (v/v) poly-l-lysine (PLL) at a concentration of 0.4 mg/ml. Further details can be found in Supplementary Note 1. The positively charged quaternary amines of PLL and negatively charged carboxylic acid residues of GOx facilitate the formation of a self-assembled hydrogel at the tip of nanopipettes. Drying-mediated self-assembly of the PLL/GOx hydrogel can then be crosslinked with glutaraldehyde vapour, Fig. 1b. Briefly, the pulled glass pipette was filled to a length of about 1 mm with a mixture of PLL and GOx by capillary action. The crosslinking reaction between PLL and GOx at the tip of the pipette was initiated and was allowed to proceed overnight in the vapour of 25% (v/v) glutaraldehyde at room temperature. Following this slow evaporation of the aqueous solution and co-condensation at the air-water interface (at the pipette tip), strong covalent crosslinking between the amino groups of PLL and GOx with the glutaraldehyde were formed, and a self-assembly nanomembrane at the tip of glass pipette was generated. Before using, the nanomembrane probes were washed with 100 mM pH 7.0 KCl to remove unreacted glutaraldehyde and any un-crosslinked reagents. The nanomembranes are very stable, and we have observed no degradation of pH stability over the duration of continuous experiments in solution (up 3 h), Supplementary Fig. 4d.

For experiments using double-barrel nanopipettes, the procedure was similar; however, one of the barrels was closed with “Blu-Tack” (Bostik, UK). The second, open barrel was kept away from sucking in PLL/GOx solution by holding a positive ~200 kPa pressure to compensate for the negative capillary force. As a result, only one barrel was modified with crosslinked PLL/GOx nanomembrane; another unmodified open barrel was used for SICM feedback control, Fig. 3a.

### Scanning ion conductance microscopy

All experiments were performed using a customised SICM setup and operated using an ICAPPIC Controller (IC-UN-001, ICAPPIC Ltd, UK). The SICM scan head consisted of a PIHera P-621.2 XY Nanopositioning Stage (Physik Instrumente, Germany) with 100 × 100 µm travel range that moved the sample and a LISA piezo actuator P-753.21 C (Physik Instrumente, Germany) with travel range 25 µm for pipette positioning along the *Z*-axis. Coarse positioning was achieved with translational stages M-111.2DG (XY directions) and M-112.1DG (*Z*-axis) (Physik Instrumente, Germany). Piezo actuators were powered by high voltage amplifiers E-503 and E-505 and servo module E-509 (Physik Instrumente, Germany). SICM control, data acquisition and analysis software were written and kindly provided by Dr Pavel Novak, ICAPPIC Ltd. Ion current was detected and recorded using a MultiClamp 700B amplifier (Molecular Devices, UK) using a 2 kHz low-pass filter. A typical external holding voltage of −200 mV was supplied to the scanning probe. The ion current and output of the capacitive sensors from all three piezo elements were monitored using an Axon Digidata 1322 A digitiser and Clampex 9.2 software (Molecular Devices, UK).

### Cyclic voltammetry and signal recording

The nanoprobe pH sensor was backfilled with 100 mM pH 7.0 KCl, contacted with an Ag/AgCl wire and immersed into a 2 mL physiological bulk solution (PBS, low-buffered solution, or buffered solution). Another Ag/AgCl electrode was placed in bulk solution acting as a reference electrode. All potentials were quoted against this reference electrode. Both electrodes were connected to a MultiClamp 700B amplifier and digitised with an Axon Digidata 1322 A (Molecular Devices) and Clampex 9.2 (Molecular Devices, UK). The potential was cycled between −0.6 V and +0.6 V vs. Ag/AgCl at a scan rate of 650 mV s^−1^. The recorded ion currents flowing in the nanoprobe were low-pass filtered at 1 kHz and analysed with pClamp 10 software (Molecular Devices). All measurements were performed in an air-conditioned room. The solution temperature was monitored throughout with a TC-344B Automatic Temperature Controller (Warner Instrument Corporation) and was maintained at 26 °C during pH experiments.

### SICM feedback-controlled pH mapping

Scanning and local pH measurements were performed with a customised SICM setup and controller (ICAPPIC Ltd., UK). For pH mapping, each pH nanoprobe was firstly calibrated with pH adjusted solutions. Then the pH-sensitive nanoprobes were typically held at −200 mV vs Ag/AgCl and used like any other standard SICM scanning probes. The SICM probe-sample distance was regulated by monitoring ion current through the nanoprobe pH sensor, which can be accurately positioned towards the cell surface under feedback control (without contact from the nanoprobe itself), and a pH measurement at each SICM topographical scanning point can be used to generate a 3D map of the pH distribution. All mapping, data acquisition and analysis software were developed by Dr Pavel Novak, ICAPPIC Ltd.

### Calibration and evaluation of the nanoprobe pH sensor

The pH-sensitive nanomembrane probes were calibrated using standard pH buffered solutions at different pH values and benchmarked against commercially available pH metre (MP 220, Mettler Toledo). Only nanoprobes that exhibited a linear response within this range was used in further studies. To test the platform, artificial H^+^ gradients were reversibly generated using tunable voltage pulses. The delivery pipettes were immersed into the PBS (pH 7.4) solution and backfilled with 100 mM HCl in order to generate an H^+^ gradient. A positive voltage of 0.5 V *vs* Ag/AgCl was applied to locally deliver H^+^, whereas, a retaining voltage of −0.5 V was applied to prevent a release and could be used as a negative control for nanoprobe pH detection. SICM feedback position control and sub-ms fast response of the Linear Piezo Stage in our SICM setup allowed for rapidly moving the pH sensor along the generated pH gradient by changing the distance from the H^+^ source. The response time was defined as the time required to reach the equilibration output of the nanoprobe.

### Double-barrel SICM-pH nanoprobe 3D pH mapping

Cells were placed on the bottom of a glass microscopy dish (35 mm diameter) and imaged using a Nikon inverted microscope. Measurements of pH on living cells were performed in low-buffered media by adding 1/4 HBSS to 137 mM NaCl, 5 mM KCl and 25 mM D-glucose (pH 7.4), which helps to maintain the pHe gradients. A schematic diagram shows the operating principles for both SICM imaging and pH being measured simultaneously, Fig. 3a. When combined with SICM, the open barrel (SICM-barrel) serves to measure the probe-cell surface distance in the same fashion of traditional SICMs. The second pH-barrel was functionalised with PLL/GOx nanomembrane to investigate the pH distribution around scanned cells. As with the single-barrel pH nanoprobe, the nanoprobe was backfilled with 100 mM KCl (pH 7.0). A typical −200 mV bias was applied to the open barrel (SICM-barrel) to induce an ion current for SICM feedback control and imaging; a typical +600 mV bias was applied to induce ion current flowing into the pH-barrel for H^+^ gradient mapping, Fig. 3c.

SICM hopping mode allows self-referencing measurements as previously used in multifunctional SECM-SICM to compare the response in bulk and near the surface at each pixel, which is particularly beneficial if the probe signal drifts over time in a living system. Our double-barrel SICM-pH nanoprobe was operating in such a self-referencing hopping mode for 3D pH mapping. Briefly, the vertical Z positioning of the hopping nanoprobe and the movement of the sample in the XY plane were controlled at a sampling frequency of 20 kHz. The double-barrel ion current was recorded using a 2 kHz low-pass filter. A four-step procedure was used to determine the height and pH at each imaging point. First, the nanoprobe was withdrawn to a distance of ~10 µm from the cell surface. Secondly, the vertical position of the nanoprobe was maintained for 1 ms, while the nanopositioning stage moved to a new imaging point in the XY plane. At this time, a reference pH of the bulk solution was measured. Thirdly, the nanoprobe was lowered at a constant fall rate of 25 nm/ms under feedback control. When the ionic current of the SICM-barrel was reduced to a set point (a drop of 0.5–1%), the Z-position was recorded for topographical imaging. The nanoprobe was then withdrawn 100 nm (to avoid any overshooting), and cell surface pH was measured for 1 ms. Fourthly, the nanoprobe was withdrawn with a specified hopping amplitude (~10 µm) from the cell surface to start a new topographical scanning and pH mapping cycle. During the pH 3D mapping, the probe pH sensing in bulk can be compared with that measurement near to the cell surface and can be used to compensate for any possible pH drift.

### Cell culture

The human malignant melanoma cell line A375M and human immortal melanocyte line Hermes 3 A were both obtained from the Wellcome Trust Functional Genomics Cell Bank (St George’s, University of London, UK). A375M cells were grown in RPMI 1640 (Gibco). Culture medium was supplemented with 10% fetal bovine serum (Sigma–Aldrich), 100 U/mL streptomycin and 100 U/mL penicillin in an atmosphere of 10% CO_2_ at 37 °C.

Hermes 3 A melanocytes were grown in RPMI 1640 medium supplemented with 10% fetal calf serum (Sigma–Aldrich), 200 nM 12-0-tetradecanoyl phorbol acetate, 200 pM cholera toxin, 10 ng/mL human stem cell factor and 10 nM endothelin 1. The Wellcome Trust Functional Genomics Cell Bank (St George’s, University of London, UK) recommends using 10% CO_2_ for growing cells of melanocytic lineage.

Jurkat-Tag cells (a gift from Prof. Stuart Niel, King’s College London) in which luciferase reporter was transfected with IL-2 promoter-luciferase plasmid and NFAT promoter-luciferase plasmid to express the large T-antigen. Jurkat-Tag cells were maintained in RPMI 1640 (Gibco) supplemented with 10% fetal bovine serum (Sigma–Aldrich) and 1% Glutamax (Gibco) at 37 °C in a humidified incubator in an atmosphere of 5% CO2. For microscopic analysis, cells were seeded in glass-bottom dishes coated with poly-l-lysine and allowed to adhere for 30 min.

MCF7 cells (obtained from the European Collection of Authenticated Cell Cultures) were infected with CD44CR1-IRES-GFP-puro lentiviral vector (Tebu-Bioscience) and selected by puromycin (0.5 μg/ml). MCF7 CD44 reporter GFP cells were maintained in Dulbecco’s Modified Eagle’s Medium (DMEM) containing 10% fetal calf serum (FCS) and 10^−8^ M estradiol (E2758 Sigma). In starved condition, the cells were maintained in phenol-red free DMEM containing 10% charcoal-stripped FSC without estradiol for 2 days. Both media were supplemented with 2 mM L-glutamine, 100 units/mL penicillin and streptomycin.

### Primary gastric parietal cell isolation and culture

Isolated rat stomach was turned inside out, and it was treated with a Ca_2+_-free DMEM containing 20 mM HEPES (pH 7.4), 0.5% BSA, 0.1 mg/ml trypsin inhibitor and 1 mg/ml Pronase E for 20 min at 37 °C. After the cell debris was removed, the stomach was further digested in DMEM containing 20 mM HEPES (pH 7.4), 0.5% BSA, 0.1 mg/ml trypsin inhibitor, 1.5 mM Pronase E, and 1 mg/ml collagenase for 30 min at 37 °C. The mixture was then filtered through a cell strainer and washed with DMEM. Isolated cells were incubated with 1 mg/ml amphotericin B for 15 min at 37 °C. After washing, the cells were loaded onto a discontinuous Optiprep (Axis-Shield) gradient. After centrifugation at 800 × *g* for 8 min, the fraction enriched in parietal cells (around 70% of the total) was collected and plated onto collagen-coated coverslips in 12-well plates and incubated at 37 °C in DMEM containing 2 mg/ml BSA, 10 mM glucose, 5 mg/ml ITES, 8 nM EGF, 5 mg/ml G418, 400 mg/ml gentamicin, 20 mg/ml novobiocin, 10 nM hydrocortisone, 100 U/ml penicillin/streptomycin and 20 mM HEPES (pH 7.4). In the experiment using the nanomembrane pH probe sensor, parietal cells were identified by using anti-H^+^, K^+^-ATPase β-subunit antibody (D032-3H, 2B6, Medical & Biological Laboratories) and diluted 1:250 Alexa Fluor 488-conjugated anti-mouse IgG antibody (ab150105, Abcam).

### pH-sensitive fluorescent dye

Fluorescein derivative 2ʹ,7ʹ-bis-(2-carboxyethyl)-5- (and-6)-carboxyfluorescein (BCECF) is the most widely used pH probe, whose excitation ratios at two different wavelengths are correlated to pH. Since BCECF does not permeate the cell membrane, and fluorescence can be measured outside the cell. BCECF (Dojindo) was chosen to evaluate the extracellular pH changes of gastric parietal cells. The fluorescence levels of BCECF around parietal cell were measured with a Leica TCS-SP5 Confocal Microscope. Z stack images were acquired using a  1 μm Z step, and the resolution was calculated to be around 0.2 μm/pixel.

### Marine diatom culture

Marine diatom *Coscinodiscus wailesii* (CCAP1013/9) was obtained from the Culture Collection of Algae and Protozoa, Scottish Marine Institute and were cultured in artificial seawater AQUIL. The diatom was grown in a controlled environmental room (illumination: 120 µmol·m^−2^·s^−1^; light/dark: 16 h/8 h; temperature: 15 °C) at Silwood Park, Imperial College London. To prepare the AQUIL, laboratory wares were acid-cleaned (>24 h in 10% HNO_3_, Fisher Scientific) and rinsed with Milli-Q water at least four times. Chemicals of ACS grade or higher purity were purchased from Sigma–Aldrich, and the medium containing synthetic ocean water and major nutrients was sterilised at 121 °C for 15 min before adding 0.2-µm filtered (polycarbonate filters, Merck Millipore Ltd.) solutions of EDTA-metals and vitamins. Diatoms were placed in a glass-bottomed microscopy dish (35 mm diameter) and observed using a Nikon inverted microscope. During the measurements, the diatoms were kept in low-buffered artificial seawater (0.5 M NaCl + 1/10 PBS, pH 7.65) exposed to light or darkness. Cells were illuminated at 200 μmol m^−2^ s^−1^ using a blue light source, and there was no change in temperature within the dish during illumination.

### Focused ion beam milling and SEM imaging

Nanomembrane probes were cut to the desired length, mounted on sample stubs and coated on all sides with 10 nm of chromium in a sputter coater (Q150T S Quorum). The probes were imaged using a FIB-SEM (CrossBeam Workstation Auriga, Carl Zeiss). Tips with the nanomembrane were located using secondary electron imaging with an accelerating voltage of 5 keV. The sensor tip was milled using a milling current of 120 pA at a working distance of 5 mm. Sections of 10 nm were ion milled and imaged by SEM at 1.6 keV, using a secondary electron in-lens detector.

### Reporting summary

Further information on research design is available in the Nature Research Reporting Summary linked to this article.

## Supplementary information

Supplementary Information

Reporting Summary

## Data Availability

The data that support the plots within this paper and other findings of this study are available from the corresponding author upon reasonable request.
