# Supplementary material for: High-resolution label-free 3D mapping of extracellular pH of single living cells
Source: Nat Commun. 2019 Dec 6;10:5610. doi: 10.1038/s41467-019-13535-1 (PMC6898398; doi:10.1038/s41467-019-13535-1)
Supplement: Supplementary file 1 — Supplementary Information [file 41467_2019_13535_MOESM1_ESM.pdf]

Supporting Information for

High-resolution label-free 3D mapping of extracellular pH of single living cells

Zhang et al.

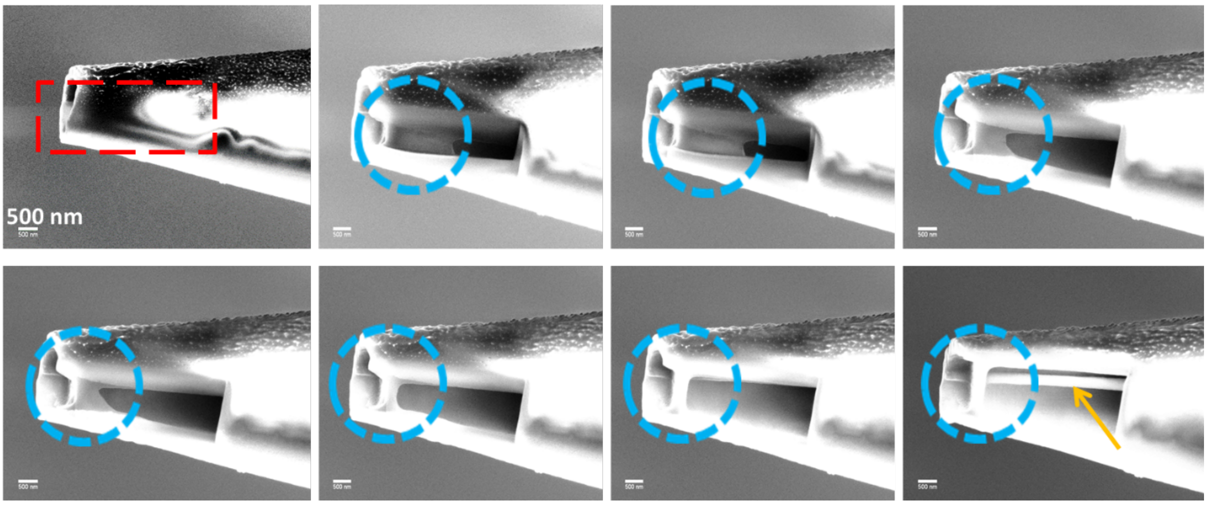

Supplementary Fig. 1| Characterisation of nanomembrane within the tip of pH probes with Focused Ion Beam Scanning Electron Microscopy (FIB-SEM). A series of cross-sectional images were taken at different stages during FIB milling to assess the thickness of the formed PLL/GOx nanomembrane inside the tip of a micropipette. The FIB milling position is marked by the dashed-rectangle (red) on the first image. The PLL/GOx nanomembrane was gradually exposed using slice-and-view in the tip of glass-pipette and is highlighted by a dashed-circle (blue). The diameter of the thin filament inside the glass pipettes (yellow arrow), can be also seen in the image in the bottom right corner. The scale bar is 500 nm.

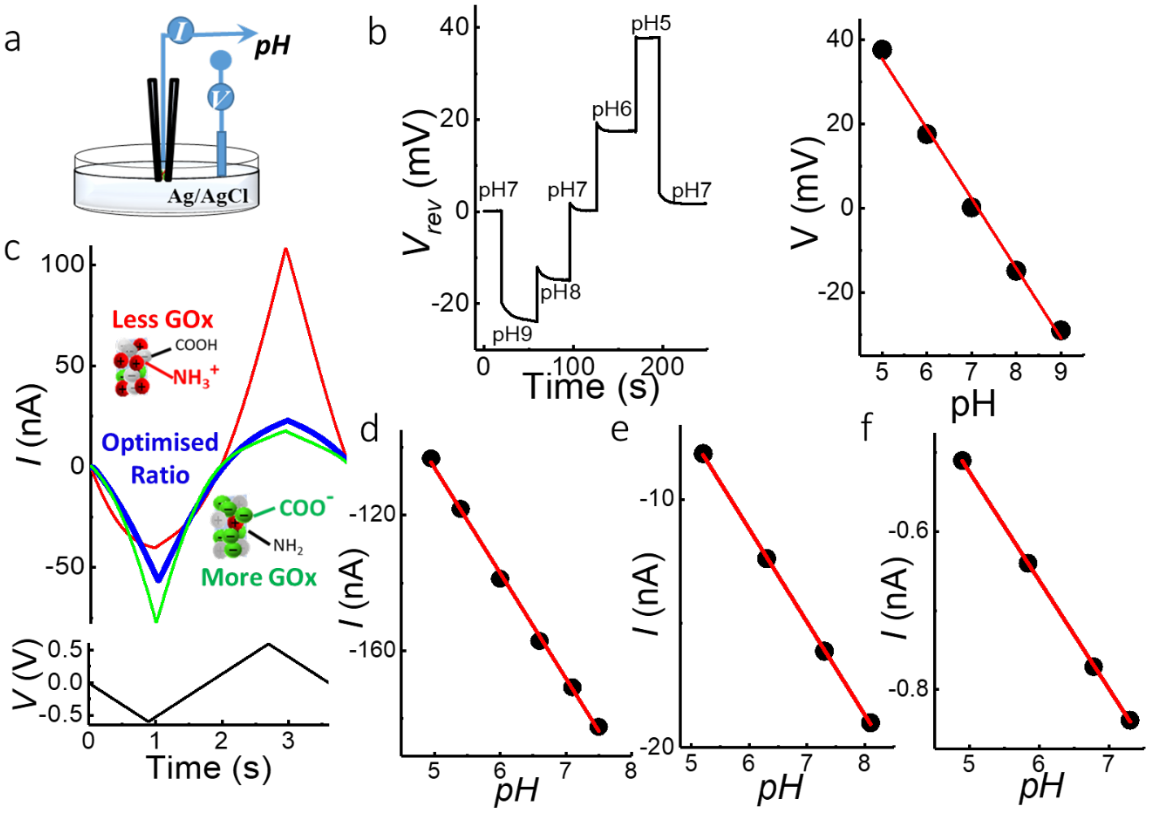

Supplementary Fig. 2| (a) Schematic of the operational principle for the pH sensor. (b) Characterisation of the nanoprobe sensor in reversal potential (V_{rev}) mode at varied pH from 5 to 9, which demonstrates linearity in pH response. (c) The surface charge of the nanomembrane can be optimally tuned by controlling the ratio of GOx to PLL. The latter can be verified by monitoring the surface charge induced ion current rectification of the nanoprobe in response to a swept voltage between −0.6 and 0.6 V. A typical holding potential of –0.6 V was used for pH sensing as shown for pipettes with different inner-diameters including a ~2.5 µm micropipette (d), a ~1 µm micropipette (e), and a ~100 nm nanopipette (f). In all cases, a linear pH response was observed.

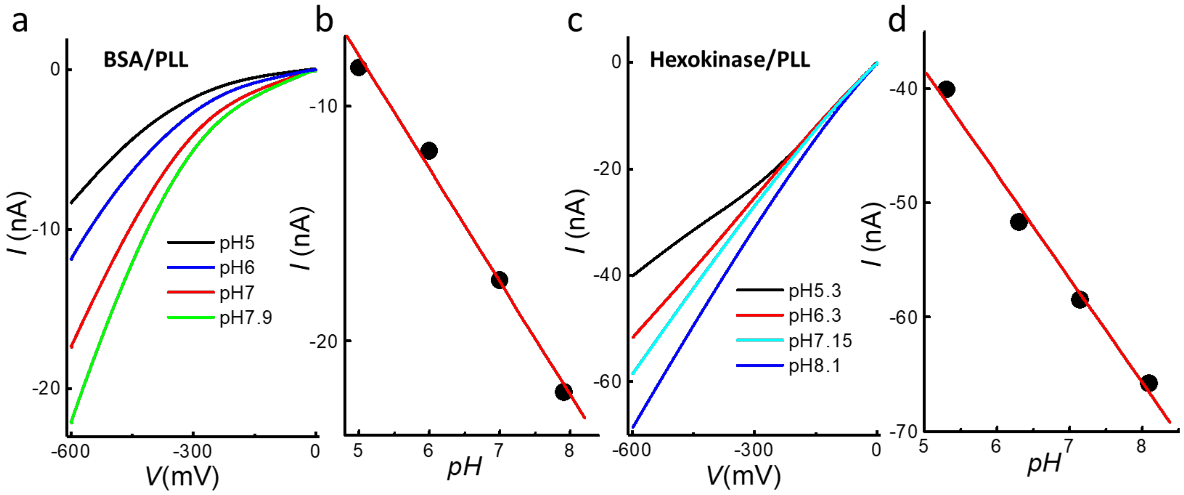

Supplementary Fig. 3| Other examples of stable zwitterionic-like nanomembranes based on PLL that were formed on a nanopore. (a) IV response at different pH for bovine serum albumin (BSA)/PLL nanomembrane and (c) IV response for Hexokinase/PLL nanomembrane. Linear current response was observed for pH between 5 and 8, at holding potential of –0.6 V for pipettes with (b) BSA/PLL nanomembrane and (d) Hexokinase/PLL nanomembrane.

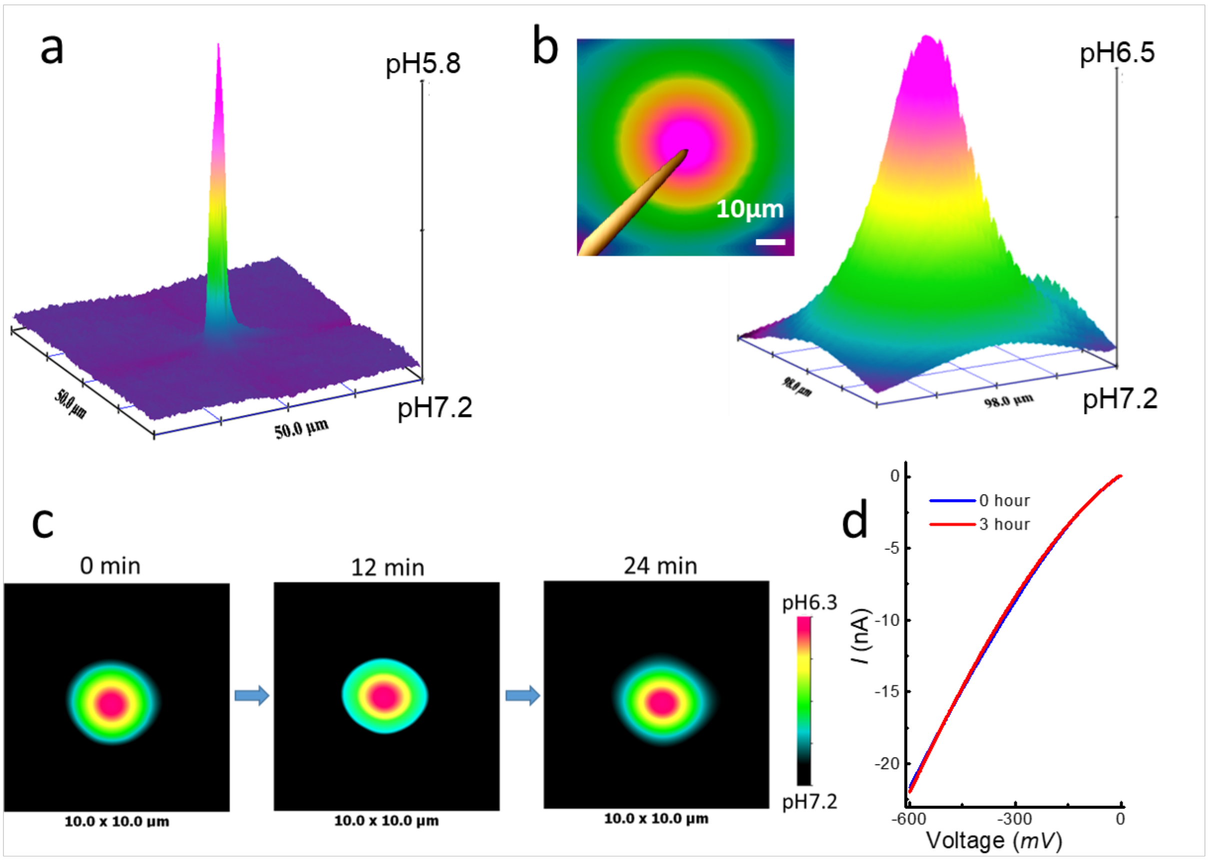

Supplementary Fig. 4| Nanoprobe sensor XY-plane 2D mapping of pH gradient generated with an H^+ supply nanopipette (a) and a micropipette (b). (c) pH mapping confirming high stability over time, using the same probe. (d) IV measurements at the beginning and after 3 hours demonstrating near-identical current response.

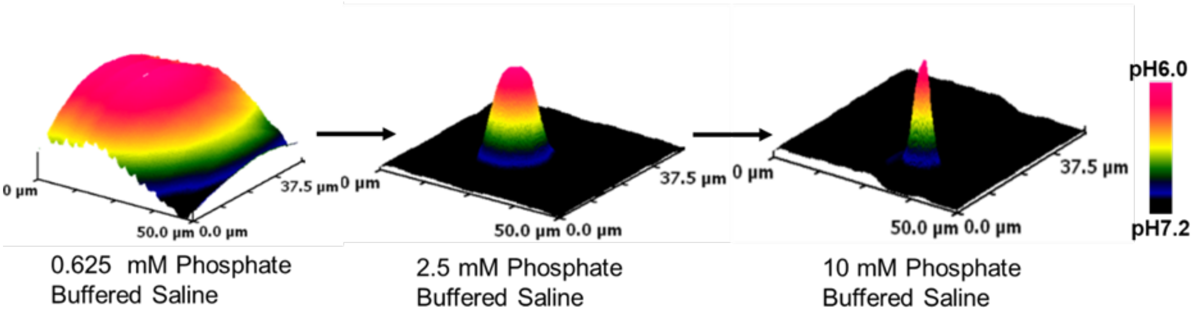

Supplementary Fig. 5| Investigation of the effects of varying buffer concentrations on the H^+ gradient with single-barrel pH sensor. A 2D mapping (in X-Y plane) of artificial nanopipette-based H^+ gradient was performed using SICM constant height mode. Initially a 0.625 mM phosphate-buffered saline was mapped out; then the effects of phosphate buffer capacities on H^+ distribution are investigated by increasing concentrations of phosphate buffer to 2.5 mM and 10 mM.

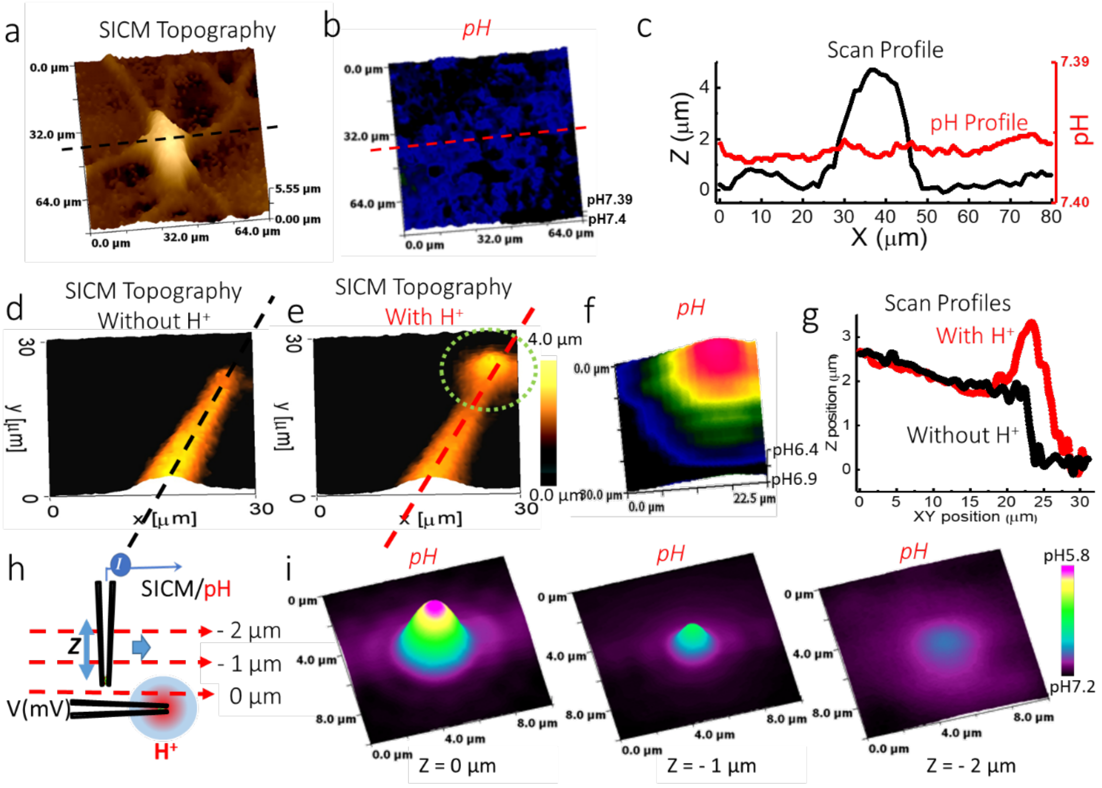

Supplementary Fig. 6| (a) SICM 3D topographical image of a low-buffered live melanocyte. (b) 3D pH mapping, done in parallel, of the same melanocyte showed no observable pH gradients. (c) Topography scanning and pH mapping profile of dotted line marked in (a) and (b). (d) SICM feedback-controlled pH-sensitive nanoprobe for topographical imaging of a glass pipette without an external supply of H^+. (e) SICM feedback-controlled pH-sensitive nanoprobe topographically imaging of the same pipette with H^+ supply. A ball-like artefact appeared with H^+ supply (highlighted with a dotted-circle). (f) Maps of H^+ gradient near the tip of the glass pipette with H^+ supply. (g) Scanning profiles of dashed-line marked on the SICM topographical image **d** (black colour) and **e** (red colour). (h) A schematic diagram showing the operation of the nanoprobe for 2D pH mapping at a controlled Z position using constant height SICM scanning mode. (i) Nanoprobe pH mapping at different Z positions relative to the H^+ supply pipette.

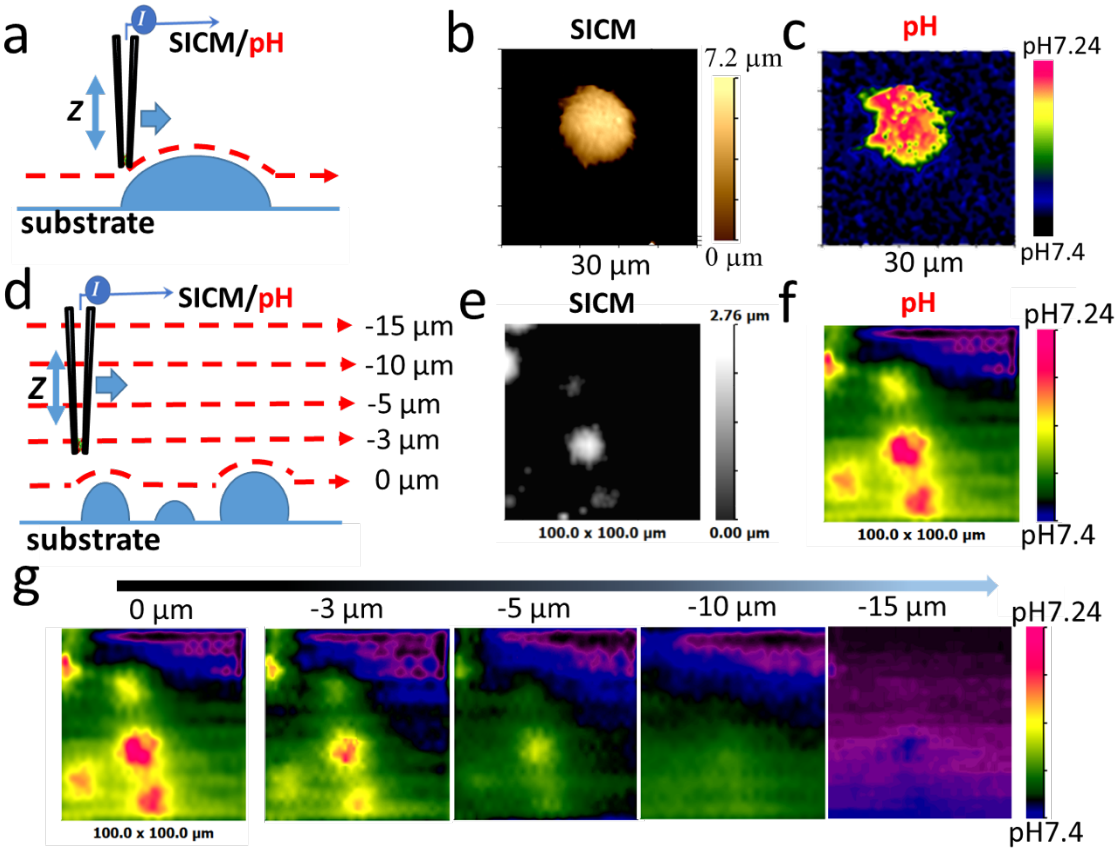

Supplementary Fig. 7| (a) The schematic shows the operation of feedback-controlled iceberg SICM mode at the single-cell level. The iceberg SICM mode controls the nanoprobe scanning and pH mapping over the top part of cells using a feedback-controlled hopping mode, but without contacting the substrate surface. Morphological scanning (b) and 3D pH gradient mapping (c) of low-buffered single living Jurkat cell are obtained simultaneously with the same nanoprobe. (d) A schematic illustrating the feedback-controlled iceberg SICM mode scanning and pH mapping for a group of cells. Using iceberg SICM scanning one can accurately estimate the cell height and position. Then the nanoprobe can be withdrawn at a set distance, and pH mapping can be performed at a constant height. A group of low-buffered melanoma cells are topographically imaged (e) and pH mapped (f) by feedback-controlled iceberg SICM mode. (g) The nanoprobe pH sensor is step withdrawn 3-15 μm away from the group of melanoma cells, then an X-Y plane 2D pHe mapping is performed at a constant height (Z position) SICM mode.

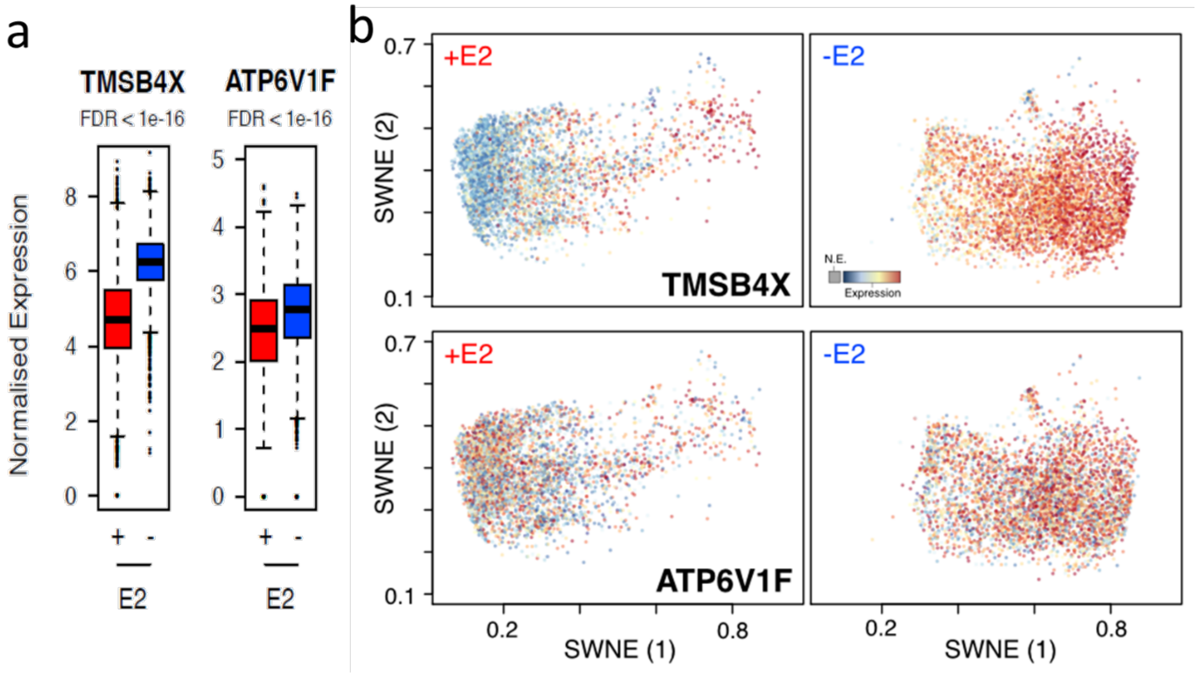

Supplementary Fig. 8| mRNA expression of TMSB4X and ATP6V1F genes in single-cell transcriptomics data of 9,980 CD44GFP-high MCF7 cells in either estradiol-supplemented (+E2) or deprived (-E2) condition. (a) Distribution of normalised expression values of TMSB4X and ATP6V1F genes from 9,980 single cells in either oestrogen-supplemented (+E2; n = 4,982) or deprived (-E2; n = 4,998) media. False discovery rate (FDR) estimated using MAST [5]. Box plots show median, interquartile values, range, and outliers (individual points). (b) Scatterplots showing a bidimensional representation of the 9,980 transcriptomes (each dot is a cell; dimensionality reduction according to similarity weighted nonnegative embedding (SWNE) [6]). The colour of each dot encodes the relative gene expression level of the indicated gene, across cells.

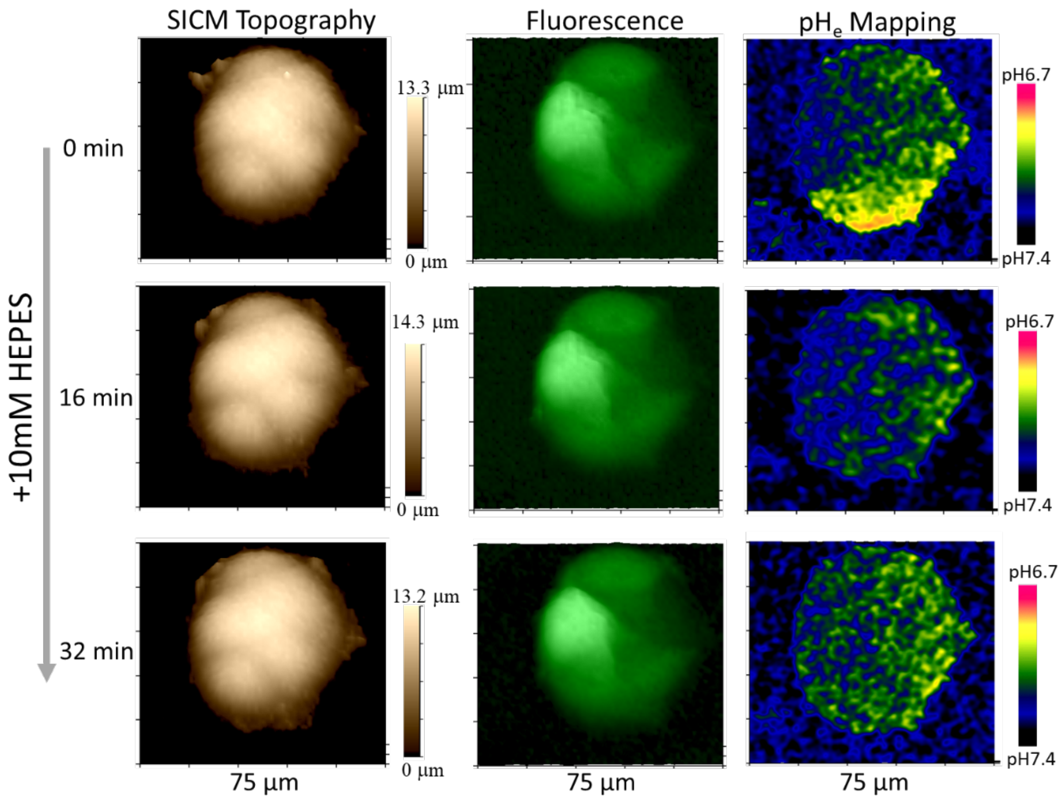

Supplementary Fig. 9| Addition of 10 mM HEPES to bulk solution markedly diminished the pHe gradient observed in living estradiol-deprived (-E2) CD44$^{GFP\text{-}high}$ MCF7 breast cancer cells (shown in Figure 3d). Continuos topographical scanning (left), CD44^{GFP} fluorescence image (middle), and 3D pHe mapping (right) of the same group of HEPES buffer-treated –E2 CD44$^{GFP\text{-}high}$ MCF7 cells with double-barrel SICM-pH nanoprobe demonstrated significant changes in pHe. The results were displayed as three rows of images from a sequence of scans recorded 16 min apart.

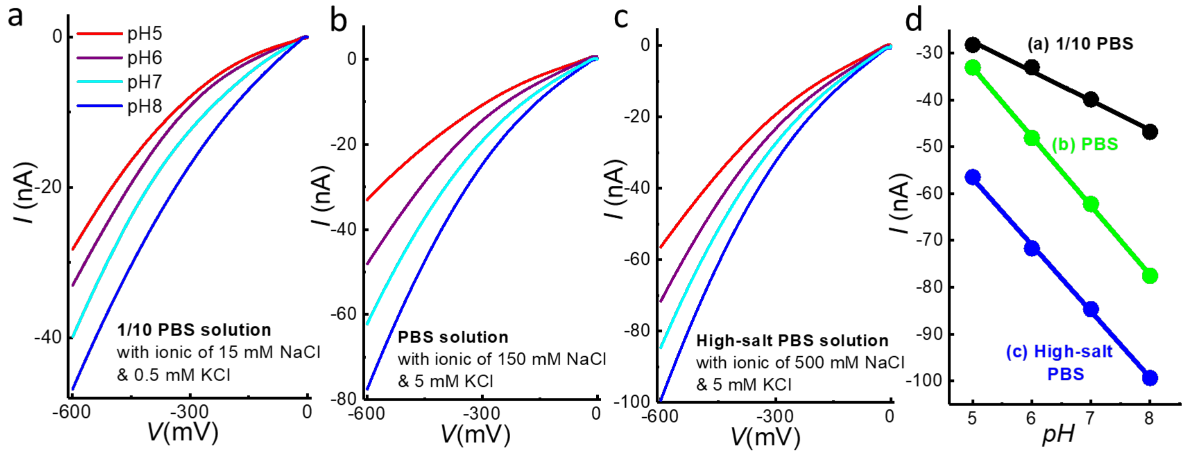

Supplementary Fig. 10| Current-voltage characteristics of the nanoprobe at different ionic strength solutions. The same nanomembrane probe was used for all experiments. The ionic strength inside the probe was kept constant at 100 mM KCl, whilst the bath solution was varied ranging from (**a**) 1/10 PBS (0.5 mM KCL and 15 mM NaCl), (**b**) PBS (5 mM KCL and 150 mM NaCl and (**c**) high-salt PBS (5 mM KCL and 500 mM NaCl). (**d**) pH-dependent ion current response of the nanoprobe at different ionic strength conditions.

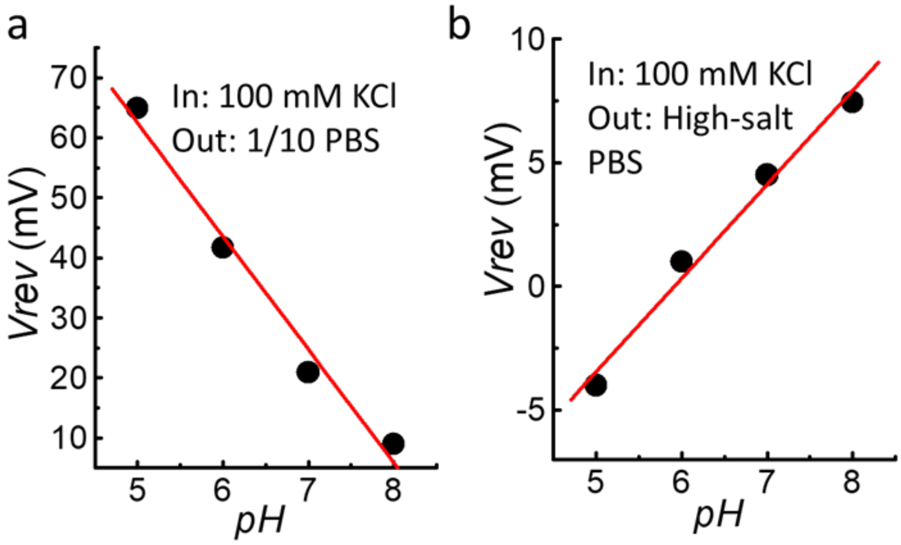

Supplementary Fig. 11| pH-dependent ion selectivity of a typical nanomembrane probe at different ionic strength conditions. The probe was characterised in reversal potential (*Vrev)* mode at pH ranging from 5 to 8. (**a)** approximately 20 mV reversal potential per pH unit was measured for 1/10 PBS solution, a value that was similar to the typical probes shown in **Supplementary Fig. 2.** (b) approximately 5 mV reversal potential per pH unit was measured in high-salt PBS solution.

Supplementary Note 1

In order to maximise the reproducibility of the membrane fabrication process, the protocol and evaluation procedures described below was used. Using such conditions, the probes exhibited very high operational stability and reproducibility as demonstrated in the repeated mapping of the generated artificial H^+ gradients. A typical probe could remain pH-sensing functional for approximately 180 days (dry shelf life at room temperature) and could be used under varying ionic strength conditions ranging freshwater to seawater as shown in Supplementary Figure 10 and 11.

1. Glass nanopipettes were pulled with a commercial P-2000 laser puller (Sutter Instrument Co.). The size and shape were shown to be highly reproducible and had a final diameter of 105 ± 11 nm (Mean ± S.D., n =10) as characterised by SEM. The pipette resistance was also highly reproducible as was determined to be 100 ± 10 MΩ (Mean ± S.D., n = 50).

2. To increase the chemical and conformational stability of GOx within the GOx/PLL nanomembrane, the protein was firstly denatured by preheating in order to decrease the size of its three-dimensional spatial conformation.

3. To ensure the nanomembrane is formed in a reproducible manner, the GOx/PLL hydrogel was filled into the nanopipette by capillary action, with solutions that had identical fluid density, viscosity, and volume.

4. Glass nanopipettes filled with the same amount and ratio of GOx/PLL hydrogel were exposed to glutaraldehyde vapour in order to facilitate a higher density of crosslinking and improved reproducibility of the drying-mediated self-assembly formation of the membrane.

5. The total current and ion current rectification ratio were used to asses membrane to membrane reproducibility rather than the absolute dimensions. For example, in a pH 7.3 solution with ~0.4 mg/ml GOx and 0.01% PLL, a rectification ratio of 0.51 ± 0.09 (Mean ± S.D., n=12) was obtained at-0.6/0.6 V. It is important to note that the ratio of GOx/PLL governs the concentration of the amino and carboxyl groups and hence pH.

Reference

1 Cao, S. *et al.* Extracellular Acidification Acts as a Key Modulator of Neutrophil Apoptosis and Functions. *Plos One* **10**, e0137221 (2015).

2 Okajima, F. Regulation of inflammation by extracellular acidification and proton-sensing GPCRs. *Cellular signalling* **25**, 2263-2271, doi:10.1016/j.cellsig.2013.07.022 (2013).

3 Douglas, E. S. *et al.* DNA-barcode directed capture and electrochemical metabolic analysis of single mammalian cells on a microelectrode array. *Lab Chip* **9**, 2010-2015 (2009).

4 Rofstad, E. K., Mathiesen, B., Kindem, K. & Galappathi, K. Acidic extracellular pH promotes experimental metastasis of human melanoma cells in athymic nude mice. *Cancer Res.* **66**, 6699-6707 (2006).

5 Finak, G. *et al.* MAST: a flexible statistical framework for assessing transcriptional changes and characterizing heterogeneity in single-cell RNA sequencing data. *Genome Biol* **16**, 278, doi:10.1186/s13059-015-0844-5 (2015).

6 Wu, Y., Tamayo, P. & Zhang, K. Visualizing and Interpreting Single-Cell Gene Expression Datasets with Similarity Weighted Nonnegative Embedding. *Cell Syst* **7**, 656-+ (2018).
