# Supplementary material for: High-resolution label-free 3D mapping of extracellular pH of single living cells
Source: Nat Commun. 2019 Dec 6;10:5610. doi: 10.1038/s41467-019-13535-1 (PMC6898398; doi:10.1038/s41467-019-13535-1)
Supplement: Supplementary file 2 — Reporting Summary [file 41467_2019_13535_MOESM2_ESM.pdf]

nature research

Corresponding author(s): Joshua B Edel

Last updated by author(s): Oct 27, 2019

Reporting Summary

Nature Research wishes to improve the reproducibility of the work that we publish. This form provides structure for consistency and transparency in reporting. For further information on Nature Research policies, see Authors & Referees and the Editorial Policy Checklist.

Statistics

For all statistical analyses, confirm that the following items are present in the figure legend, table legend, main text, or Methods section.

n/a	Confirmed	
☐	☒	The exact sample size (n) for each experimental group/condition, given as a discrete number and unit of measurement
☐	☒	A statement on whether measurements were taken from distinct samples or whether the same sample was measured repeatedly
☐	☒	The statistical test(s) used AND whether they are one- or two-sided<br>*Only common tests should be described solely by name; describe more complex techniques in the Methods section.*
☒	☐	A description of all covariates tested
☒	☐	A description of any assumptions or corrections, such as tests of normality and adjustment for multiple comparisons
☐	☒	A full description of the statistical parameters including central tendency (e.g. means) or other basic estimates (e.g. regression coefficient) AND variation (e.g. standard deviation) or associated estimates of uncertainty (e.g. confidence intervals)
☐	☒	For null hypothesis testing, the test statistic (e.g. F, t, r) with confidence intervals, effect sizes, degrees of freedom and P value noted<br>*Give P values as exact values whenever suitable.*
☒	☐	For Bayesian analysis, information on the choice of priors and Markov chain Monte Carlo settings
☒	☐	For hierarchical and complex designs, identification of the appropriate level for tests and full reporting of outcomes
☒	☐	Estimates of effect sizes (e.g. Cohen's d, Pearson's r), indicating how they were calculated

Our web collection on statistics for biologists contains articles on many of the points above.

Software and code

Policy information about availability of computer code

Data collection	Data were collected with MultiClamp 700B amplifier and digitised with an Axon Digidata 1322A (Molecular Devices) and Clampex 9.2 (Molecular Devices); scanning and local pH measurements were collected with SICM setup and controller (ICAPPIC Ltd., UK).
Data analysis	Data were analysed with pClamp 10 software (Molecular Devices); pH mapping and analysis was done using SICM software (ICAPPIC Ltd., UK).

For manuscripts utilizing custom algorithms or software that are central to the research but not yet described in published literature, software must be made available to editors/reviewers. We strongly encourage code deposition in a community repository (e.g. GitHub). See the Nature Research guidelines for submitting code & software for further information.

Data

Policy information about availability of data

All manuscripts must include a data availability statement. This statement should provide the following information, where applicable:

- Accession codes, unique identifiers, or web links for publicly available datasets
- A list of figures that have associated raw data
- A description of any restrictions on data availability

All raw data that support the figures within this paper are available from the corresponding author on reasonable request

Field-specific reporting

Please select the one below that is the best fit for your research. If you are not sure, read the appropriate sections before making your selection.

☒ Life sciences ☐ Behavioural & social sciences ☐ Ecological, evolutionary & environmental sciences

For a reference copy of the document with all sections, see nature.com/documents/nr-reporting-summary-flat.pdf

Life sciences study design

All studies must disclose on these points even when the disclosure is negative.

Sample size	Sample size no formal methods were used, but sample sizes employed in this study are consistent with previously published works. Single-cell transcriptomics data of all 9980 individual MCF7 cells; pH mapping and detecting from a single or a group of individual cells
Data exclusions	No data excluded
Replication	All attempts at replication were successful (a minimum of 3 and more typically 4-8 times)
Randomization	No randomization was used. The procedures employed required a sequential procedure. For example, nanopipettes were fabricated, sample was measured and finally analyzed.
Blinding	Blinded experiments design was not required.

Reporting for specific materials, systems and methods

We require information from authors about some types of materials, experimental systems and methods used in many studies. Here, indicate whether each material, system or method listed is relevant to your study. If you are not sure if a list item applies to your research, read the appropriate section before selecting a response.

Materials & experimental systems

n/a	Involved in the study	
☐	☒	Antibodies
☐	☒	Eukaryotic cell lines
☒	☐	Palaeontology
☒	☐	Animals and other organisms
☒	☐	Human research participants
☒	☐	Clinical data

Methods

n/a	Involved in the study	
☒	☐	ChIP-seq
☒	☐	Flow cytometry
☒	☐	MRI-based neuroimaging

Antibodies

Antibodies used	anti-H+, K+-ATPase β-subunit antibody (D032-3H, 2B6, Medical & Biological Laboratories) and Alexa Fluor 488-conjugated anti-mouse IgG antibody (ab150105, Abcam).
Validation	Antibodies were validated by the suppliers. https://www.mblbio.com/bio/g/dtl/A/?pcd=D032-3H https://www.abcam.com/donkey-mouse-igg-hl-alexa-fluor-488-ab150105.html

Eukaryotic cell lines

Policy information about cell lines

Cell line source(s)	Human breast cancer MCF7 cells were obtained from ECACC;The human malignant melanoma cell line A375M and human immortal melanocyte line Hermes 3A were obtained from the Wellcome Trust Functional Genomics Cell Bank (St George's, University of London, UK).
Authentication	Cell lines were authenticated by the suppliers
Mycoplasma contamination	All cell lines tested negative for mycoplasma contamination
Commonly misidentified lines (See ICLAC register)	No any commonly misidentified lines used in this study
